# *Nkx2-5* Loss of Function in the His-Purkinje System Hampers Its Maturation and Leads to Mechanical Dysfunction

**DOI:** 10.3390/jcdd10050194

**Published:** 2023-04-27

**Authors:** Caroline Choquet, Pierre Sicard, Juliette Vahdat, Thi Hong Minh Nguyen, Frank Kober, Isabelle Varlet, Monique Bernard, Sylvain Richard, Robert G. Kelly, Nathalie Lalevée, Lucile Miquerol

**Affiliations:** 1 Aix-Marseille Univ, CNRS UMR 7288, IBDM, 13288 Marseille, France; caroline.choquet@univ-amu.fr (C.C.); juliette.vahdat@univ-amu.fr (J.V.); nguyen-thi-hong.minh@usth.edu.vn (T.H.M.N.); robert.kelly@univ-amu.fr (R.G.K.); 2 INSERM, CNRS, Université de Montpellier, PHYMEDEXP, 34295 Montpellier, France; pierre.sicard@inserm.fr (P.S.); sylvain.richard@inserm.fr (S.R.); 3 Aix-Marseille Univ, INSERM UMR 1090, TAGC, 13288 Marseille, France; nathalie.lalevee@univ-amu.fr; 4 Aix-Marseille Univ, CNRS, CRMBM, 13385 Marseille, France; frank.kober@univ-amu.fr (F.K.); isabelle.varlet@univ-amu.fr (I.V.); monique.bernard@univ-amu.fr (M.B.); 5 Aix-Marseille Univ, INSERM UMR 1263, C2VN, 13005 Marseille, France; 6 Aix-Marseille Univ, INSERM, MMG, 13385 Marseille, France; 7 Department of Life Sciences, University of Science and Technology of Hanoi, Vietnam Academy of Science and Technology, Hanoi 10072, Vietnam

**Keywords:** His-Purkinje system, fast conduction markers, RSR’ complex, *Cx40*, cell dropout, strain defects, cardiac dyssynchrony

## Abstract

The ventricular conduction or His-Purkinje system (VCS) mediates the rapid propagation and precise delivery of electrical activity essential for the synchronization of heartbeats. Mutations in the transcription factor *Nkx2-5* have been implicated in a high prevalence of developing ventricular conduction defects or arrhythmias with age. *Nkx2-5* heterozygous mutant mice reproduce human phenotypes associated with a hypoplastic His-Purkinje system resulting from defective patterning of the Purkinje fiber network during development. Here, we investigated the role of *Nkx2-5* in the mature VCS and the consequences of its loss on cardiac function. Neonatal deletion of *Nkx2-5* in the VCS using a *Cx40-CreERT2* mouse line provoked apical hypoplasia and maturation defects of the Purkinje fiber network. Genetic tracing analysis demonstrated that neonatal *Cx40*-positive cells fail to maintain a conductive phenotype after *Nkx2-5* deletion. Moreover, we observed a progressive loss of expression of fast-conduction markers in persistent Purkinje fibers. Consequently, *Nkx2-5*-deleted mice developed conduction defects with progressively reduced QRS amplitude and RSR’ complex associated with higher duration. Cardiac function recorded by MRI revealed a reduction in the ejection fraction in the absence of morphological changes. With age, these mice develop a ventricular diastolic dysfunction associated with dyssynchrony and wall-motion abnormalities without indication of fibrosis. These results highlight the requirement of postnatal expression of *Nkx2-5* in the maturation and maintenance of a functional Purkinje fiber network to preserve contraction synchrony and cardiac function.

## 1. Introduction

Electrical impulses delivered by specialized components of the conduction system (CS) ensure the rhythm and the coordination of cardiac contractions [1,2]. Electrical activity initiates in the sinoatrial node (SAN) and then reaches the atrioventricular node (AVN), the unique electrical connection with the ventricles in the definitive heart. From the AVN, the electrical activity takes a fast-conducting route mediated by the His-Purkinje or ventricular conduction system (VCS). The VCS comprises the His or AV bundle (AVB), right and left bundle branches, and ends in a complex network of Purkinje fibers (PFs). The VCS represents only 1–2% of the cardiac volume but is responsible for the rapid cardiac conduction essential for the normal rhythm of cardiac contractions [3,4]. The electrophysiological properties of these specialized conductive cardiomyocytes result from the expression of a large range of specific genes coding for ion channels enabling active conduction, and gap junctions responsible for passive conduction velocity and a low level of contractile proteins [5,6,7,8]. Altered cardiac conduction can lead to ventricular arrhythmias and bundle branch blocks (BBB), which are associated with increased mortality in heart failure patients due to myocardial dyssynchrony [9]. In addition, terminal conduction delay of left ventricular depolarization, showing abnormal QRS complex but different from either RBBB or LBBB, causes major ventricular arrhythmias due to impaired tissue surrounding old infarct scars [10].

*Nkx2-5* encodes for an essential homeobox transcription factor that orchestrates cardiac development [11]. In humans, mutations of *NKX2-5* with a high penetrance often promote the occurrence of severe and progressive AV conduction blocks and atrial septal defects [12]. In transgenic mice, *Nkx2-5^+/−^* embryos and mice progressively reproduce the phenotype observed in human patients, including atrial septal defects and conduction disturbances [13]. Electrocardiogram analysis reveals that *Nkx2-5* haploinsufficient mice present a prolonged PR interval and a progressive elongation of the QRS. This phenotypic evolution is associated with progressive loss of the AVB [14,15]. Indeed, adipose tissue has been found to replace the AVB in a human patient with an *NKX2-5* mutation [15]. Moreover, patients carrying mutations in *NKX2-5* present a large spectrum of congenital heart diseases frequently associated with conduction disturbances or arrhythmias, and long-term follow-up reveals a high incidence of sudden cardiac death in these patients with aging [16,17]. However, the underlying mechanisms remain unclear. In particular, whether *Nkx2-5* plays a primary role within conductive cardiomyocytes remains an open question.

We have previously shown that *Nkx2-5* plays a crucial role in Purkinje network patterning in mice by controlling the progressive recruitment of PF during ventricular development [18,19]. In the present study, we characterized the role of *Nkx2-5* in the mature VCS. We created a conditional knockout of *Nkx2-5* within the VCS using a tamoxifen-inducible Cre mouse line [20,21]. Exploiting this strategy, we removed *Nkx2-5* in the VCS without affecting *Nkx2-5* expression in the contractile or working ventricular myocardium. *Nkx2-5* VCS conditional deletion at birth provoked a mild VCS hypoplasia and progressive loss of expression of fast-conduction markers, which resulted in the appearance of an altered ventricular activation sequence. Longitudinal analysis revealed that these conduction defects were associated with ventricular dyssynchrony. *Nkx2-5* VCS-deleted mice developed contractility defects and early diastolic dysfunction associated with a decreased ejection fraction. This study reveals a cell-autonomous role for *Nkx2-5* in maintaining a functional conductive phenotype in the VCS and protecting against heart dysfunction.

## 2. Materials and Methods

### 2.1. Mouse Lines

The investigation was approved by the ethics committee for animal experimentation of the French ministry (no. 01055.02). Animal procedures conformed to the guidelines from Directive 2010/63/EU of the European Parliament for the Care and Use of Laboratory Animals. *Cx40-CreERT2*, *R26-YFP* and *Nkx2-5-floxed* mouse lines were genotyped as previously reported [20,21,22]. To conditionally delete *Nkx2-5*, tamoxifen was injected intraperitoneally into newborn pups (P0 or P1) in a single injection (10 µL). Tamoxifen (T-5648, Sigma) was dissolved at a concentration of 20 mg/mL in ethanol/sunflower oil (10/90). *Nkx2-5-*∆*Neo^fl/fl^::Cx40^Cre/+^* were designated as *Nkx2-5*^∆*VCS*^ mice and compared to control mice *Nkx2-5^+/+^::Cx40^Cre/+^*, both receiving Tam injection at birth. 

### 2.2. Macroscopic and Histological Analyses

Mice were euthanized by cervical dislocation and hearts from young and old animals were rapidly collected in cold PBS (1×) at 2 and 12 months. Macroscopic examination of the internal surface of the ventricles was performed as previously described [23]. Whole-mount and histological immunofluorescence and image analysis were carried out as previously described [19]. Antibodies used in this study are specific to *Nkx2-5* (Sc8697 Santa-Cruz, Dallas, TX, USA), GFP (AbD Serotec, Purchheim, Germany), RFP (Rockland, Pottstown, PA, USA), Contactin-2 (AF1714 R&D system, Minneapolis, MN, USA), Pecam-1 (MEC13.3-BD Pharmingen, San Jose, CA, USA), HCN4 (Millipore, Burlington, MA, USA), ETV-1 (Abcam, Cambridge, U.K.), Troponin I (Abcam) and WGA-Cy3 (Sigma, St. Louis, MO, USA). Antibodies against Cx43 and *CX40* are homemade and previously described [6,23].

### 2.3. Cardiac Magnetic Resonance Imaging (MRI)

MRI was carried out every 2 months on the same animal groups of 2–12-month-old mice under isoflurane anesthesia (4% for the induction and 2% during the image recordings). The experiments were performed on a Bruker Biospec Avance 4.7 T/30 imager (Bruker Biospin GmbH, Ettlingen, Germany), and images were analyzed as previously described [19].

### 2.4. Echocardiography

Echocardiography was performed for two groups of mice (3 and 12 months old) under isoflurane anesthesia (2%) using a Vevo 2100 ultrasound system (VisualSonics, Toronto, Canada) equipped with a real-time micro-visualization scan head probe (MS-550D) operating at a frame rate of 300 frames per sec (fps). Left ventricular (LV) characteristics were quantified according to the standards of the American Society of Echocardiology and the Vevo 2100 Protocol-Based Measurements and Calculations guide, as previously described [20]. High-frequency speckle tracking for strain analysis was performed on parasternal long-axis B-mode loops. The standard deviation of time to peak strain corrected by the inter-beat interval was used to measure the intraventricular dyssynchrony [24]. LV strain analysis was performed offline using Vevostrain analysis software 3.1.1 (VisualSonics). To quantify the peak longitudinal strain rate during early LV filling, the “reverse peak” option was used.

### 2.5. Surface Electrocardiography

Surface ECGs were performed on anesthetized mice. An induction with 5% isoflurane was followed by maintenance at 2% in a constant flow of oxygen at 1 L/min. ECGs were recorded every two months from 2 to 12 months using a bipolar system as previously described [19].

### 2.6. Statistical Analysis

Data are expressed as means ± standard error of the mean (SEM) for bar graphs and median with min./max. for box plots. Significant differences between groups were determined using one-way or two-way analysis of variance (ANOVA) followed by Sidak post hoc testing with PGraphPad Prism software (PGraphPad Prism 7.0, La Jolla, CA, USA). A *p* value < 0.05 was considered statistically significant.

## 3. Results

### 3.1. Conditional Deletion of Nkx2-5 in the Ventricular Conduction System

**Figure 1:**
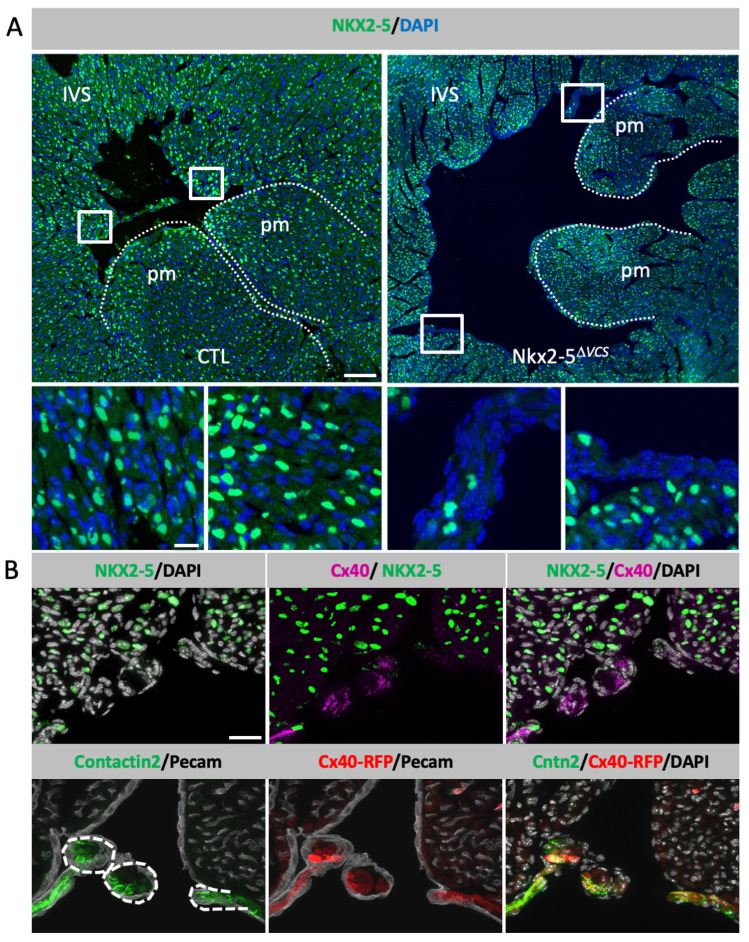
Condition deletion of *Nkx2-5* in the ventricular conduction system. (**A**) *Nkx2-5* immunofluorescence on transversal sections of P10 hearts from control (CTL) and *Nkx2-5*^∆*VCS*^ mutant mice. High magnification of the squares is represented below. IVS: interventricular septum; pm: papillary muscles. Scale bars = 100 µm; high-magnification image bar = 25 µm. (**B**) Co-immunofluorescence of *Nkx2-5* and *Cx40* or CNTN2 and *Cx40*-RFP on serial sections of P10 *Nkx2-5*^∆*VCS*^ mutant hearts. Pecam1 and Dapi staining were used to delineate cardiac contours and nuclei. Scale bar = 50 µm.

To conditionally delete *Nkx2-5* gene in the VCS, we crossed mice containing *Floxed-Nkx2-5-*∆*neo* alleles with *Cx40-CreERT2* mice. Cre recombinase activity was induced by tamoxifen injection into newborn pups, thereafter designated as *Nkx2-5*^∆*VCS*^. The efficiency of *Nkx2-5* deletion was verified by immunofluorescence on sections from control and *Nkx2-5*^∆*VCS*^ mice at P10. *Nkx2-5*-negative cardiomyocytes are restricted to subendocardial cells, representing a very low percentage of cells in the entire heart (Figure 1A). Using Contactin-2 (Cntn2) to identify cells of the VCS [25], we found that *Nkx2-5*-negative cells co-localize with Cntn2-positive cells (Figure 1B). These *Nkx2-5*-negative cardiomyocytes are also positive for *Cx40* expression detected using a *Cx40* antibody or through the expression of the *Cx40-RFP* allele (Figure 1B). As shown previously, a single postnatal injection of tamoxifen is sufficient to target a large proportion of cardiomyocytes of the VCS [21] and efficiently delete *Nkx2-5* in these cells.

### 3.2. Neonatal Loss of Nkx2-5 in the VCS Disturbs Its Maturation and Provokes Apical PF Hypoplasia

**Figure 2:**
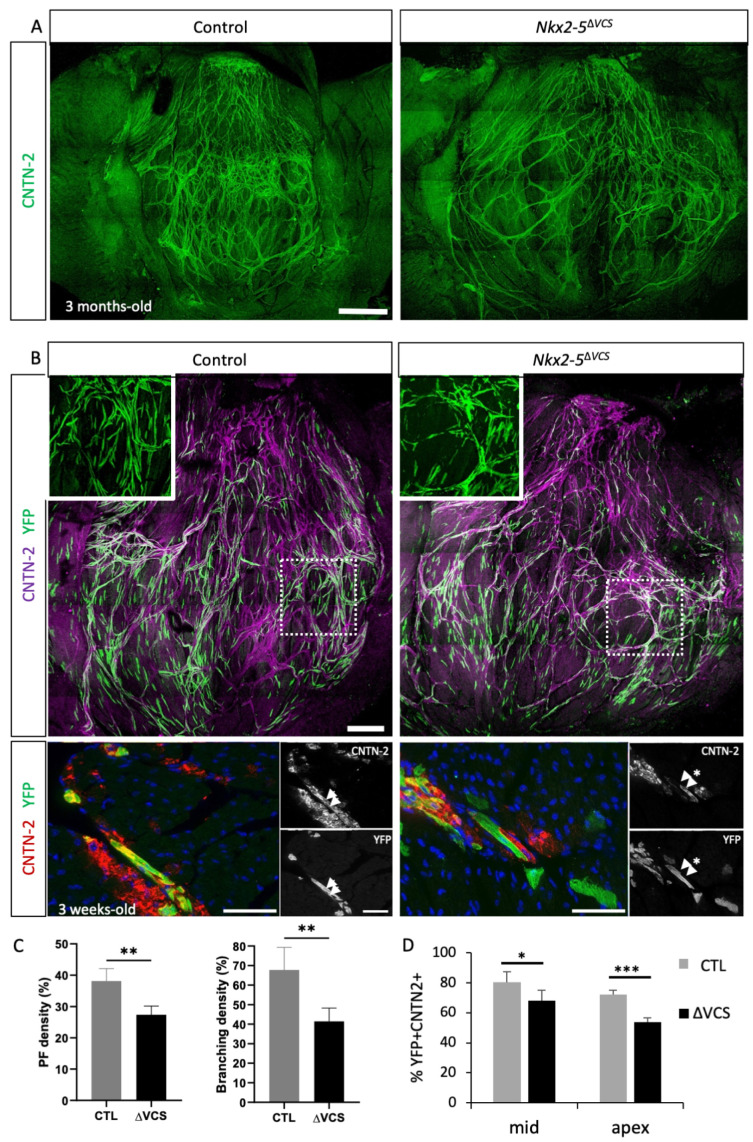
*Nkx2-5* VCS-conditional deletion mice present PF hypoplasia by cell dropout. (**A**) Whole-mount immunofluorescence with Contactin-2 on opened LV from 3-month-old control and *Nkx2-5*^∆*VCS*^ hearts. Scale bar = 1 mm. (**B**) Genetic tracing of ventricular *Cx40*-positive cells after Cre induction at E18.5 in control and *Nkx2-5*^∆*VCS*^ mice showing the distribution of YFP+ cells in the PF network indicated by a co-immunofluorescence with CNTN2 at P20. High magnifications of peripheral PFs are presented in inserts. Scale bar = 0.5 mm. Below are sections from genetic tracing experiments stained with YFP and CNTN2 antibodies. Scale bars = 50 µm. (**C**) Quantification of PF density and branching density from images treated with angiotool. (**D**) Quantification of the percentage of YFP+ cells included in the VCS. N = 3 hearts per group. Mean ± SD; Student *t*-tests: * *p* < 0.05; ** *p* < 0.01; *** *p* < 0.001 *Nkx2-5*^∆*VCS*^ vs. control.

To investigate the phenotypic consequences of the loss of *Nkx2-5* in the Purkinje fiber (PF) network, we performed whole-mount immunofluorescence using Cntn2 antibody on opened left ventricles from 3-month-old control and *Nkx2-5*^∆*VCS*^ mice (Figure 2A). *Nkx2-5*^∆*VCS*^ mutant mice present a reduced density of the PF network compared to control mice (Figure 2C), a defect that is more pronounced in the apical region (Figure 2A). Loss of *Nkx2-5* in the VCS thus provokes a mild hypoplasia of PF, primarily affecting the apical part of the left ventricle. In contrast, prior work has shown that earlier deletion during development results in severe VCS hypoplasia [19].

To understand the cellular mechanism responsible for this PF hypoplasia, we performed a genetic tracing analysis of *Nkx2-5*-deleted cardiomyocytes to follow their fate. We crossed *Cx40-CreERT2::Nkx2-5^fl/+^* with *Rosa26-YFP::Nkx2-5^fl/+^* mice and we induced Cre activity by tamoxifen injection into pregnant females at E18.5. We performed whole-mount immunofluorescence with Cntn-2 to compare the localization of YFP-positive cells in hearts from 3-week-old *Nkx2-5^+/+^* (WT) and *Nkx2-5*^∆*VCS*^ mice (Figure 2B). *Cx40*-derived (YFP-positive) cardiomyocytes are present in the same proportion in WT and *Nkx2-5*^∆*VCS*^ mutant hearts, demonstrating that those cells are not lost after *Nkx2-5* deletion. In contrast to the situation in the control hearts, a large number of these YFP cells, mainly localized in the apex, do not express the conductive marker Cntn2, indicative of a maturation defect towards a conductive phenotype. We quantified this on sections and counted the percentage of YFP+ cells integrated in the VCS with the co-expression of Cntn-2 (Figure 2D). Only 50% of YFP+ cells co-expressed Cntn-2 in the apex of *Nkx2-5*^∆*VCS*^ mutant hearts compared to 70% in WT hearts (Figure 2D). The majority of YFP+/Cntn-2- cells were close to VCS (Cntn-2+) cells and maintained an elongated shape characteristic of PF cells (Figure 2B). This analysis demonstrates a failure of *Nkx2-5*-negative cardiomyocytes to robustly maintain a conductive phenotype and suggests that PF hypoplasia in *Nkx2-5*^∆*VCS*^ mutant hearts may arise by a mechanism of cell dropout.

### 3.3. Persistent Nkx2-5-Negative Conductive Cells Progressively Downregulate Fast-Conduction Markers

To further analyze the cellular phenotype of the cardiac conduction system after conditional deletion of *Nkx2-5*, we carried out immunostaining on sections from 3-month-old control and *Nkx2-5*^∆*VCS*^ mutant hearts. *Nkx2-5* was not deleted in the AVN where *Cx40-Cre* was not expressed and only partially in the AVB (Figure S1). These two components expressed a high level of Hcn4, ETV1, and Cntn-2 in both control and *Nkx2-5*^∆*VCS*^ mutant hearts. The central part of the conduction system is thus largely unaffected in *Nkx2-5*^∆*VCS*^ mutant mice.

**Figure 3:**
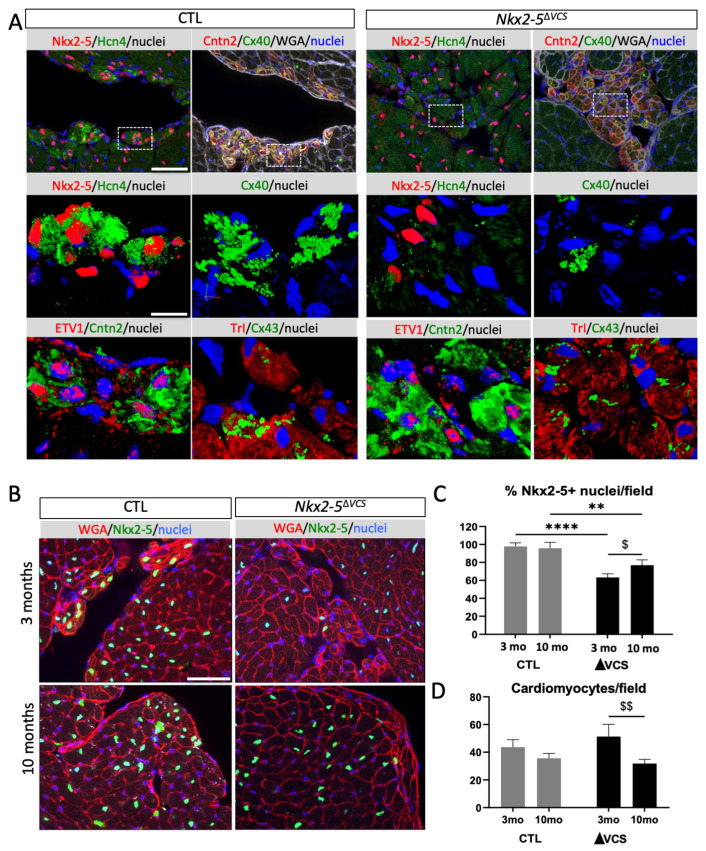
Ventricular conduction defects in *Nkx2-5*^∆*VCS*^ mutant hearts. (**A**) Immunofluorescence with *Nkx2-5* and HCN4 or ETV1 and Contactin-2 or *Cx40*, CNTN2 and WGA or Troponin I (TrI) and Cx43 on serial sagittal sections at the level the left Purkinje fibers from control (CTL) and *Nkx2-5*^∆*VCS*^ hearts. Scale bar = 100 µm. High magnifications of the selected area (rectangle) are presented below. Scale bar = 20 µm. (**B**) High magnifications at the level of LPF stained with WGA-cy3 and *Nkx2-5* from 3- and 10-month-old control (CTL) and *Nkx2-5*^∆*VCS*^ hearts. Scale bar = 50 µm. (**C**) *Nkx2-5* deletion was quantified by counting the percentage of *Nk2-5*-positive cardiomyocytes per frame at the subendocardial surface of transverse sections from 3- and 10-month-old control (CTL) and *Nkx2-5*^∆*VCS*^ (∆VCS) mice. (**D**) Quantification of cardiomyocyte hypertrophy by counting the number of cardiomyocytes per frame on high-magnification images of transverse sections from 3- and 10-month-old control (CTL) and *Nkx2-5*^∆*VCS*^ (∆VCS) mice. *n* = 20–30 frames per heart; N = 3 mice per group; mean ± SD; two-way analysis of variance (ANOVA) followed by Sidak post hoc tests: ** *p* < 0.01; **** *p* < 0.0001 ΔVCS vs. control; $ *p* < 0.05; $$ *p* < 0.01, 3-month-old vs. 10-month-old hearts.

In the peripheral VCS, numerous Cntn-2+/*Nkx2-5-* cells were observed in *Nkx2-5*^∆*VCS*^ mutant hearts (Figure 3A). However, only very few of these Cntn-2+/*Nkx2-5-* cells express *Cx40* and Hcn4; in contrast, these cells continue to express the conductive markers ETV1 and Cx43. Overall, these results show that the deficit of *Nkx2-5* in the VCS induces the loss of a subset of markers important for electrical conduction such as *Cx40* and Hcn4 but not ETV1 or Cntn-2, in line with the idea that *Nkx2-5* promotes a conductive phenotype by directly controlling the expression of specific genes.

With aging, the density of Cntn-2 and *Cx40* double-positive cells was very low in 10-month-old *Nkx2-5*^∆*VCS*^ mutant heart sections (Figure S2). High-magnification views showed a drastic reduction in these two markers in *Nkx2-5*^∆*VCS*^ mutant compared to the control hearts while the expression of Cx43 was preserved. Using WGA-Cy3 staining to label cell contours, we quantified the number of total cardiomyocytes per section and the percentage of *Nkx2-5+* cells (Figure 3B). Quantification revealed a similar number of cardiomyocytes per field in control and *Nkx2-5*^∆*VCS*^ mutant mice, suggesting the absence of hypertrophy in these mutants. However, a reduced number of cardiomyocytes per field in old mice suggests a slight increase in cardiomyocyte size developing with age in both control and *Nkx2-5*^∆*VCS*^ mutant mice. Moreover, the thin contour of WGA staining surrounding cardiomyocytes excludes any signs of fibrosis in both control and *Nkx2-5*^∆*VCS*^ mutant hearts. Quantification of the percentage of *Nkx2-5+* cardiomyocytes per field revealed an increase in *Nkx2-5*^∆*VCS*^ mutant mice between 3 and 10 months of age, increasing from 60% to 75%. These data indicate that, in addition to premature cell dropout, a loss of *Nkx2-5*-deleted cardiomyocytes naturally occurs with age. Moreover, the loss of fast-conduction markers suggests that persistent PFs in *Nkx2-5*^∆*VCS*^ mice are less conductive.

### 3.4. Conditional Deletion of Nkx2-5 in the VCS Leads to Cardiac Functional Defects

**Figure 4:**
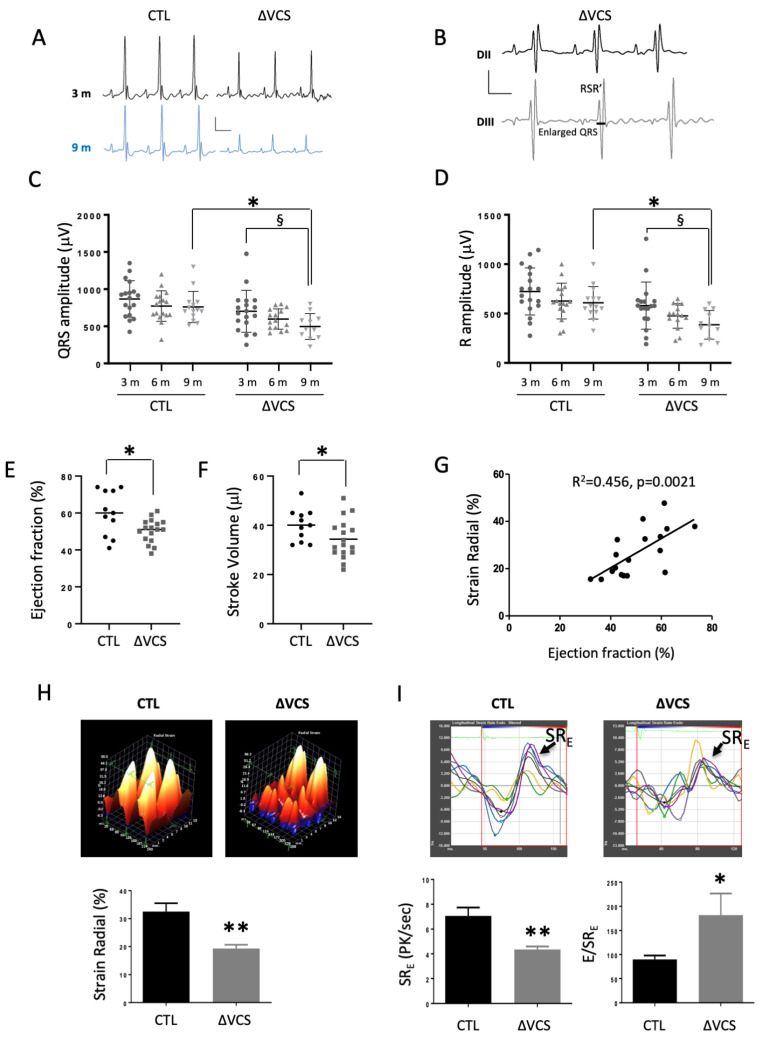
*Nkx2-5* deletion in the conduction system of neonate mice leads to QRS defects associated with ventricular dysfunction. (**A**) Representative tracings from surface ECG measured in lead II in anaesthetized mice at 3 and 9 months old. In control mice, QRS complexes present a classic shape. Tracings from *Nkx2-5*^Δ*VCS*^ mice demonstrated ventricular conduction defects. (**B**) Representative tracings of a QRS pattern with RSR’ shape observed in *Nkx2-5*^Δ*VCS*^ mice. (**C**,**D**) Graphs representing the evolution of the QRS and R amplitudes measured in the same mice over a year. QRS and R amplitudes are lower in the *Nkx2-5^ΔVCS^* group compared to the control (N = 14–18 in the control and N = 12–18 in *Nkx2-5*^Δ*VCS*^). Mean ± SD; two-way analysis of variance (ANOVA) followed by Sidak post hoc tests: * *p* < 0.05ΔVCS vs. control; § *p* < 0.05 3-month-old vs. 9 month-old hearts. (**E**,**F**) Graphs representing MRI measurements of the ejection fraction (EF) and stroke volume (SV) in 6-month-old mice. Both parameters are significantly decreased in *Nkx2-5*^Δ*VCS*^ mice (N = 11 in control and N = 17 for *Nkx2-5*^Δ*VCS*^). Mean ± SD; Student *t*-tests: * *p* < 0.05 *Nkx2-5*^Δ*VCS*^ vs. control. (**G**) Measurements of cardiac parameters by echocardiography in 10-month-old control and *Nkx2-5*^Δ*VCS*^ mice show a strong correlation between ejection fraction and strain radial. Mean ± SD; Student *t*-tests: ** *p* < 0.01 *Nkx2-5*^Δ*VCS*^ vs. control. (**H**) Strain radial is decreased in 10-month-old *Nkx2-5*^Δ*VCS*^ in comparison to control mice. (**I**) Representative curves of longitudinal strain rate in 10-month-old control and *Nkx2-5*^∆*VCS*^ mice. Diastolic function estimates by measurements of SR_E_ and E/SR_E_. Mean ± SD; Student *t*-tests: * *p* < 0.05; ** *p* < 0.01 *Nkx2-5*^Δ*VCS*^ vs. control.

Surface electrocardiograms (ECGs) were recorded in control and *Nkx2-5*^∆*VCS*^ mutant mice to investigate cardiac electrical activity at 3, 6 and 9 months of age. ECG traces revealed a small increase in QRS complex duration in *Nkx2-5*^∆*VCS*^ mutant mice, which was significant at 6 months (Table 1). This increase was not representative of the defects observed in some *Nkx2-5*^∆*VCS*^ mutant mice. Indeed, the QRS complex had an RSR*’* shape in some mutant mice, the number of which increased with age from 28% at 3 months to 55% at 9 months (Table 1, Figure 4B). In these mice with an abnormal QRS complex, the average QRS duration was 3 ms longer than in the control mice. In addition, the amplitudes of the QRS complexes in derivation II were smaller in the mutant mice (Table 1, Figure 4A). This difference increased with aging and was due to the decrease in the amplitude of the R-wave (Figure 4C,D). No QRS defects were observed in control mice, and in contrast to ventricular activation, the repolarization phase, represented by QT intervals and T wave amplitude, and atrial activation, represented by PR interval, were unaffected in both groups of mice (Table 1).

The cardiac function of individual 6-month-old mice was evaluated using cardiac MRI. Cardiac parameters calculated from MRI analysis showed a decrease in the ejection fraction (EF) and stroke volume (SV) in *Nkx2-5*^∆*VCS*^ mutant compared to control mice (Figure 4E,F). No differences were observed in morphological parameters between both groups or with aging (Table 2, Figure S3).

To better characterize this cardiac phenotype, we used a highly sensitive speckle-tracking-based strain imaging technique to detect regional ventricular wall deformation. *Nkx2-5*^∆*VCS*^ mutant mice present reduced radial strain predominantly affecting the anterior wall region (Figure 4H). We found a strong correlation between reduced EF and radial strain (Figure 4G).

In addition, high-frequency speckle-tracking echocardiography was used to access subtle changes in diastolic function. Representative curves of longitudinal strain rate showed an important defect in the coordination of myocardial deformation and a reduced peak global strain rate during early diastole (SR_E_) in *Nkx2-5*^∆*VCS*^ mutant compared to control mice (Figure 4I). The diastolic index E/SR_E_ was increased in *Nkx2-5*^∆*VCS*^ mutant mice. Taken together, these data suggest a diastolic dysfunction in old mutant mice (Figure 4I). LV mechanical dyssynchrony was determined as time-to-peak variation, defined as the standard deviation of time to peak over all six segments. Control mice presented minimal longitudinal dyssynchrony (8.0 ± 0.5% at 3 months and 8.5 ± 1.8% at 10 months), whereas *Nkx2-5*^∆*VCS*^ mice had significant dyssynchrony (13.9 ± 1.4% at 3 months and 14.9 ± 1.9% at 10 months) compared to age-matched control mice (*p* < 0.05 at 3 months and *p* < 0.01 at 10 months). Defects of myocardial deformation and systolic/diastolic dysfunction suggest that premature VCS cell dropout and the loss of fast-conduction markers progressively lead to conduction defects associated with wall-motion abnormalities and ventricular dysfunction.

## 4. Discussion

In this study, we analyzed the cardiac function of mice lacking *Nkx2-5* expression in the ventricular conduction system after conditional deletion in *Cx40*-positive cardiomyocytes one day after birth. Our data demonstrate that *Nkx2-5* deletion restricted to the VCS induces progressive ventricular conduction defects that result in early intraventricular dyssynchrony followed by mechanical strain defects and decreased EF. *Nkx2-5* is required in *Cx40*-positive cells to maintain a PF phenotype. *Nkx2-5*^∆*VCS*^ mutant hearts present a significant PF hypoplasia and loss of expression of a subset of conduction markers. These results highlight the importance of *Nkx2-5* in the maturation and maintenance of the PF network and the role of loss of function of VCS cells in promoting conduction defects, ventricular mechanical dyssynchrony and left ventricular dysfunction.

Previous studies have shown an important role for *Nkx2-5* in cardiac function in human patients as well as in *Nkx2-5* haploinsufficient mice [26,27,28]. To date, the role of *Nkx2-5* has been mainly studied in contractile cardiomyocytes, which represent the force-generating myocardial component of the heart. Deletion of Nkx2-5 disturbed the expression of numerous cardiac genes, inducing calcium-handling defects and decreased contractibility, which impaired cardiac function [29,30,31]. Here, *Nkx2-5* deletion was restricted to a very small population of cells forming the VCS, representing only 1–2% of the total heart volume. ECG analysis revealed progressively reduced QRS amplitude and abnormal wave patterns, with RSR’ complex associated with a wide QRS, in line with morphological and histological disturbance of the PF network in *Nkx2-5*^∆*VCS*^ mutant mice. In particular, the drastic decrease in R wave amplitude corresponding to a cutback of the parietal vector [32] is consistent with the hypoplasia of PF in the median and apical part of the myocardium. Mouse models in which *Cx40* is abolished had a remodeling of passive conductance properties and developed prolonged intervals for all ECG parameters, including split QRS complexes, in line with uncoordinated ventricular activation [33,34,35]. Moreover, spatial propagation of electrical activity through the working myocardium, reflected by the mean frontal plane axis, is abnormal in *Cx40*^−/−^ mice [34]. Our data on *Nkx2-5* deletion in the VCS are consistent with these results and now confirm the role of a *Cx40*-deficient VCS in the occurrence of altered ventricular activation sequence [33,34,36,37].

*Nkx2-5* regulates numerous cardiac genes involved in active conduction, such as *HCN4*, encoding the channel responsible for the I_f_ current, and *SCN5A*, encoding the sodium channel Nav1.5 [31,38]. Regarding the role of HCN4 in PF [39], its decreased expression may also participate in the *Nkx2-5*^∆*VCS*^ phenotype, particularly the abnormal QRS pattern because of disturbed active ventricular conduction. Conversely, *Nkx2-5*-deleted conductive cardiomyocytes maintain expression of ETV1, Cx43 and Cntn-2, suggesting that these genes are not direct targets for this transcription factor. These data are consistent with *Nkx2-5* operating downstream of ETV1, a transcription factor responsible for the activation of a rapid conduction genetic program [40]. PF hypoplasia and reduced expression of *Nkx2-5* and fast-conduction markers including *Cx40* and Nav1-5 have been recently observed in cardiomyocyte-specific deletion of ETV1 [41]. Besides this intrinsic role of *Nkx2-5* in the maintenance of a fast-conductive PF phenotype, we observed cell dropout of a number of *Nkx2-5*-negative elongated cardiomyocytes associated with the loss of Cntn-2 expression. In patients, it was shown that the RSR’ complex associated with a wide QRS, a unique mural conduction defect unrelated to right or left bundle branch block, was a sign of myocardial infarct scarring [10]. Our data reveal that loss of VCS cells and their fast conduction capacity is similar to the terminal conduction delay of left ventricular depolarization within impaired tissue due to myocardial infarction [10]. These data show that *Nkx2-5* is required postnatally to retain cells in the VCS during maturation of the PF network and during aging. This provides new mechanistic insights into the previously defined requirement for maximal *Nkx2-5* levels in VCS development [42].

*Nkx2-5*^∆*VCS*^ mice present conduction disturbances and ventricular dyssynchrony associated with LV dysfunction. Indeed, these mutant mice develop strain defects, which are correlated with a decreased EF and diastolic dysfunction. However, these mice exhibited no structural changes in the myocardium and developed no apparent signs of cardiac hypertrophy or fibrosis, known triggers of heart failure. In this respect, abnormal conduction and progressive mechanical dysfunction would result from intrinsic properties of the VCS rather than a secondary consequence of other cardiac defects. Our data strongly suggest a direct role for conduction defects in the appearance of cardiac dysfunction. In a previous study, we demonstrated that the conditional deletion of *Nkx2-5* in trabecular cells during embryonic development provokes a hypertrabeculated phenotype associated with cardiac hypertrophy, subendocardial fibrosis and severe VCS hypoplasia, which induced heart failure with age [19]. However, young mice developed LV dysfunction detected by a reduction in EF similar to *Nkx2-5*^∆*VCS*^ mice. Together, these data suggest that conduction defects represent a triggering factor for LV dysfunction in non-compaction cardiomyopathy and might explain the poor prognostic of patients with non-compaction cardiomyopathy associated with ECG defects [43,44,45]. Furthermore, our results highlight the important role of the ventricular conduction system in the origin of conduction defects and cardiac dysfunction in non-compaction cardiomyopathy. Likewise, the asymptomatic phenotype of numerous individuals with hypertrabeculated hearts may arise from a different etiology in which the VCS is not affected [46].

Finally, this is the first study highlighting the direct effect of conduction defects on contractile function. Using recently developed technology, including high-frequency speckle tracking echocardiography [24], we were able to detect mechanical dyssynchrony in *Nkx2-5*^∆*VCS*^ hearts. Mechanical dyssynchrony measures the variation in the timing of regional ventricular deformation during cardiac contraction and is altered in mouse models with contractile function defects or with age [47,48]. One of the explanations for age-related dyssynchrony is increased fibrosis and lipid content [49,50]; however, in our mutants, the defects were restricted to the Purkinje fiber network. Recent studies have described dyssynchrony as an important prognostic factor in patients with heart diseases for cardiac resynchronization therapy (CRT) [51]. CRT is now an important therapy for heart failure patients with reduced EF and ventricular conduction delay [52]. However, not all patients respond to this treatment, and those who do have more severe systolic dyssynchrony, suggesting that the VCS may be affected [53,54]. Our data strongly suggest that conduction defects are an important trigger of mechanical dyssynchrony that can be corrected by CRT and provide predictive insights into this responsiveness. If the VCS rather than the contractile compartment is primarily affected, as in our mutant mice, CRT may be more efficient. This assumption warrants further investigation in the future to establish clear diagnostic criteria for CRT.

## Figures and Tables

**Table 1:** Surface ECG parameters.

	3 Months Old		6 Months Old		9 Months Old		2-Way ANOVA	
Groups	Ctrl	∆VCS	Ctrl	∆VCS	Ctrl	∆VCS	Age	Group
N	18	18	17	15	14	11		
Heart Rate (BPM)	519 ± 83	507 ± 61	493 ± 63	490 ± 54	495 ± 69	504 ± 57	NS	NS
PR (ms)	31.8 ± 3.3	32.0 ± 3.6	34.3 ± 4.3	33.3 ± 3.2	34.4 ± 3.3	33.0 ± 3.1	¶	NS
QRS lead III (ms)	12.3 ± 1.3	13.4 ± 2.8	11.6 ± 1.1	13.6 ± 2.8 *	11.7 ± 1.5	13.3 ± 3.3	NS	¶¶
RSR’-QRS (ms)		15.6 ± 3.4 (28%)		15.9 ± 2.5 (47%)		15.2 ± 3.0 (55%)		
QT (ms)	41.7 ± 1.7	42.8 ± 4.0	42.7 ± 5.3	43.8 ± 7.2	47.5 ± 7.7 §	45.6 ± 8.2	¶	NS
T (µV)	65.6 ± 34.2	63.9 ± 38.1	78.2 ± 34.5	70.7 ± 21.3	87.7 ± 25.5	63.6 ± 25.3	NS	NS
QRS (µV)	868.1 ± 245.3	702.7 ± 282.0	772.1 ± 205.3	597.7 ± 136.7	760.1 ± 209.6	497.2 ± 174.1 *,§	¶	¶¶¶¶
S (µV)	144.9 ± 72.1	122.2 ± 85.6	144.8 ± 91.1	120.8 ± 82.3	150.8 ± 96.5	110.9 ± 62.9	NS	NS
R (µV)	723.3 ± 238.0	580.6 ± 238.8	627.2 ± 181.1	476.9 ± 124.4	609.3 ± 164.8	386.3 ± 144.9 *,§	¶¶	¶¶¶¶

N = number of animals. RSR’-QRS = averaged QRS value only for traces with an RSR’ M-shape. All parameters were recorded in lead II except for QRS in lead III as indicated. Data are expressed as mean ± SD. Two-way analysis of variance (ANOVA) followed by Sidak post hoc tests: * *p* < 0.05 ΔVCS vs. control; § *p* < 0.05 vs. 3 months old of the same group; ¶ *p* < 0.05; ¶¶ *p* < 0.01; ¶¶¶¶ *p* < 0.0001.

**Table 2:** cMRI parameters.

	6 Months Old	
Groups	Ctrl	∆VCS
* Physiological parameters*		
N	11	17
Body weight (g)	46.5 ± 7.7	43.1 ± 8.6
Heart rate (BPM)	466 ± 61	503 ± 47
*Morphological parameters*		
EDV (μL)	67.6 ± 9.4	68.2 ± 13.1
ESV (μL)	27.9 ± 11.3	33.9 ± 7.7
LV mass (mg)	100.4 ± 11.5	95.9 ± 19.5
LV mass sys (mg)	121.4 ± 18.3	118.2 ± 26.4
sWTn (%)	41.4 ± 18.3	36.8 ± 10.6
*Functional parameters*		
EF (%)	60 ± 12	50 ± 6 *
SV (μL)	40.1 ± 6.6	34.4 ± 8.0 *

N = number of animals. EDV, end-diastolic volume, and ESV, end-systolic volume, represent the internal volume of the left ventricle at the end of the diastole or systole; LV mass and LV mass sys represent the mass of the left ventricular wall at the end of the diastole or systole; sWTn, systole wall thickening, represents the thickening of the left ventricular wall at the end of the systole compared to the end of the diastole; EF, ejection fraction, represents the percentage of volume measured at the end of the diastole expulsed at the end of the systole; SV, stroke volume, represents volume of blood expulsed at the end of the systole. Data are expressed as mean ± SD, two-way analysis of variance (ANOVA) followed by Sidak post hoc tests: * *p* < 0.05 ΔVCS vs. control.

## Data Availability

Data are available from the authors upon request.

## Supplementary Materials

The following supporting information can be downloaded at: https://www.mdpi.com/2308-3425/10/5/194/s1, Figure S1: Normal AVN and AV bundle in *Nkx2-5*^∆*VCS*^ mutant hearts; Figure S2: PF hypoplasia is aggravated in 10-month-old *Nkx2-5*^∆*VCS*^ mice; Figure S3: Short-axis cine images recorded by MRI at end-diastole (DIA) and end-systole (SYS).
