# Supplementary material for: Nkx2-5 Loss of Function in the His-Purkinje System Hampers Its Maturation and Leads to Mechanical Dysfunction
Source: J Cardiovasc Dev Dis. 2023 Apr 27;10(5):194. doi: 10.3390/jcdd10050194 (PMC10219388; doi:10.3390/jcdd10050194)
Supplement: Supplementary file 1 [file jcdd-10-00194-s001.zip › jcdd-2354505-supplementary.pdf]

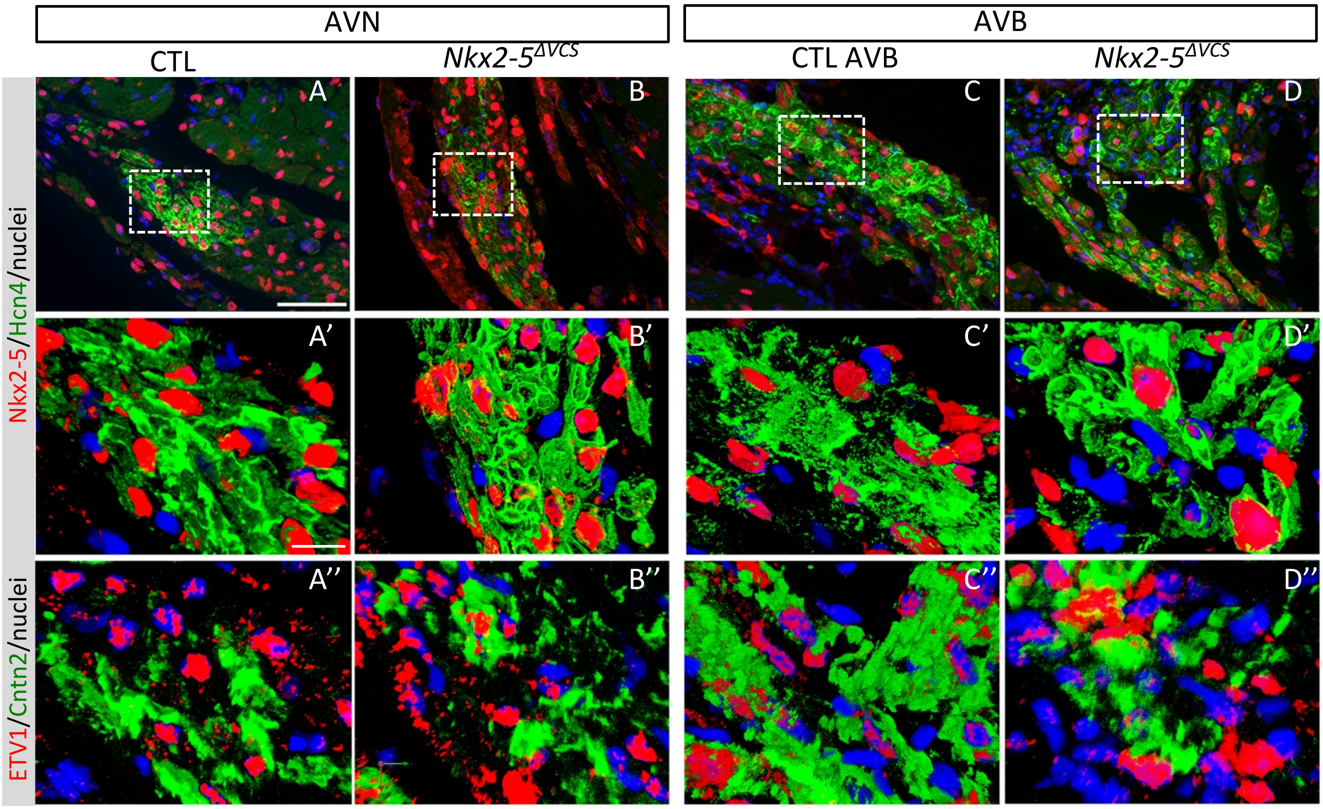

Figure S1: Normal AVN and AV bundle in *Nkx2-5$^{\Delta VCS}$* mutant hearts.
(A-D) Immunofluorescence with Nkx2-5 and HCN4 or ETV1and Contactin-2 on serial sagittal sections at the level of the atrioventricular node (AVN) (A-B) or atrioventricular bundle (AVB) (C-D) from control (Ctrl), *Nkx2-5$^{\Delta VCS}$* hearts. High magnifications are presented in A'-D' and A"-D". Scale bar= 100µm; 20µm in high mag images.

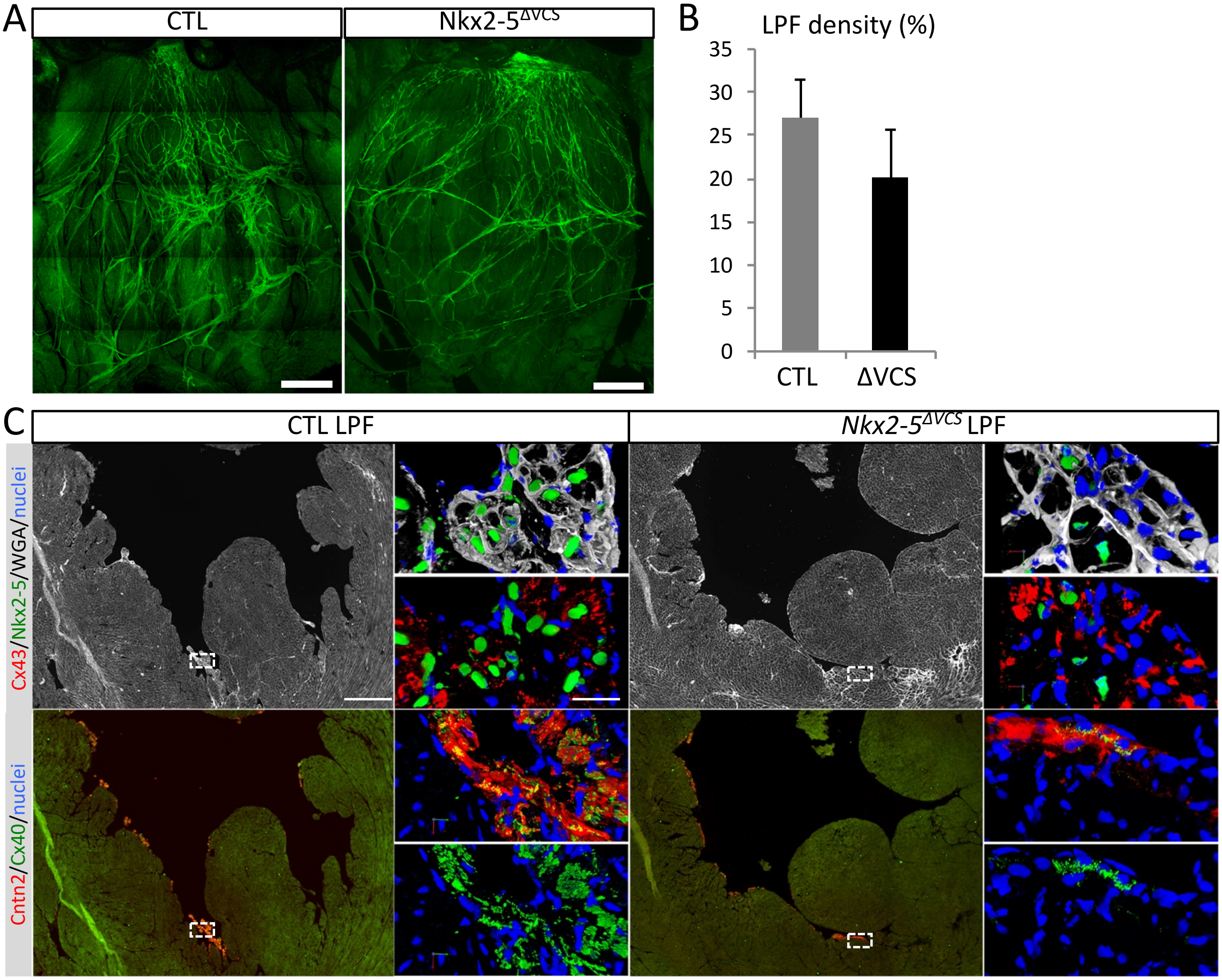

Figure S2: PF hypoplasia is aggravated in 10 month-old *Nkx2-5$^{\Delta VCS}$* mice.
(A) Whole-mount immunofluorescence with Contactin-2 on opened LV from 10 month-old control and *Nkx2-5$^{\Delta VCS}$* hearts. Scale bar = 1mm (B) Quantification of LPF density by Angiotool. (C) Immunofluorescence with Nkx2-5, Cx43 and WGA or Contactin-2 and Cx40 on serial sagittal sections at the level of the left Purkinje fibers from 10 month-old control (CTL) and *Nkx2-5$^{\Delta VCS}$* hearts. Scale bar= 200µm; 25µm in high mag images.

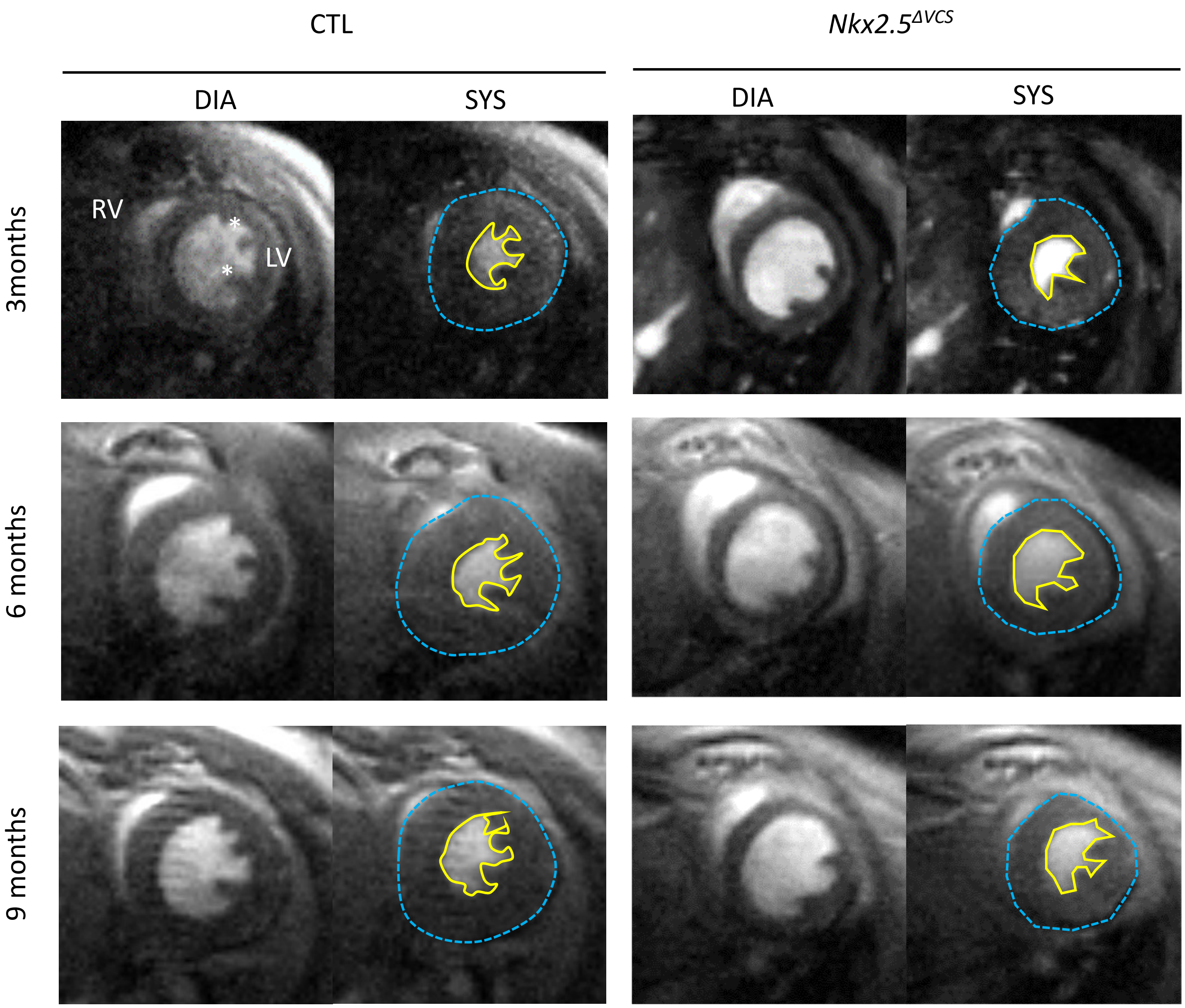

Figure S3 :Short-axis cine images recorded by MRI at end-diastole (DIA) and end-systole (SYS).

Images represent the follow-up of the same heart per mutant groups at 2-4, 6-8 and 9-10 month-old, with the yellow line indicating the endocardial outline and the blue line indicating the epicardial outline.
